# Filariasis—I

**Published:** 1908-05-02

**Authors:** George C. Low

**Affiliations:** Lecturer in Tropical Diseases. West London Hospital.


### Tropical Diseases.

### FILARIASIS.—I.

By GEORGE C. LOW, M.A., M.B., Lecturer in Tropical Diseases, West London Hospital.

The term filariasis has been adopted to indicate in man and other animals an invasion by different members of the filaridæ, a group of nematoid worms. Man may be infested by at least four kinds, while horses, cattle, dogs, birds, and other animals have species of their own. In many instances the parasite and host live in complete harmony, but, on the other hand, the worms may set up different lesions which may or may not be detrimental to the host. In the dog, for example, there is a filaria, technically termed the *Filaria immitis*, which inhabits the right side of the heart; when the numbers become excessive the circulation may be so seriously embarrassed that death from cardiac failure results.

Adult filariæ are always bisexual; the females give birth to viviparous embryos which escape from the host in various ways, directly in the case of *Filaria medinensis* (the guinea worm), indirectly through the blood in the case of *Filari bancrofti*, *Filaria immitis*, and others, suctorial insects extracting them from this medium. As far as we know the embryos of all the different species must undergo a metamorphosis in some intermediate host. To understand their somewhat complicated life-history it is essential to remember the different sites in which the adults and embryos occur. The human parasites are often termed blood filariæ because of the presence of their larval or embryonic forms in the circulating blood, but one must always clearly understand that the adults which give rise to these are hidden away in some of the inner tissues of the body, the exact situation depending on the species under consideration. Another difficulty to contend with is that in many instances the adult and embryonic forms were given different names before the relation of the two forms was known.

### Filaria Bancrofti.

Having explained some of these facts, one may next pass to a description of the common filariæ of man, taking first those which pour their embryos into the blood. Of these by far the most important, because it often gives rise to pathological lesions and symptoms, is the *Filaria bancrofti.*

The embryo of this form was discovered before the parents were known and it was first called the *Filaria sanguinis hominis*; later, when it became apparent that other species might also be detected in man's blood, the name was changed to the *Filaria nocturna* because it appears in the blood by night. The correct terminology for this parasite is now: adult, *Filaria bancrofti;* embryo, *Microfilaria bancrofti vel nocturna.*

The habitat of the adult forms of this species is the lymphatic system—the thoracic duct, for example, the pelvic lymphatics, or those of the axilla, or the more superficial ones of the limbs. They are thin like fine catgut; measure from two to two and a half inches in length; are, of course, quite visible to the naked eye; may be coiled up in a mass together, or may lie more free. The female after fertilisation pours embryos into the lymph in large numbers; these are only $\frac{1}{90}$ inch in length and therefore only visible under the microscope. These larval or embryonic forms are carried by the lymph stream, *via* the thoracic duct, to the blood, and once they gain this medium they neither develop nor change in form until they are sucked up by suitable mosquitoes. If, therefore, in examining an individual's blood we find such parasites under the microscope, it simply means that the individual is the subject of filariasis and has adult *Filaria bancrofti* in his interior. The young, as far as we know, are harmless; the adults only are capable of setting up disease. Inhabiting as they do the lymphatic trunks, it is clear that their pathological effects will be connected with this system. It does not necessarily follow, however, that pathological lesions will ever appear; they may or they may not, depending on a variety of circumstances. The only way, therefore, to determine what percentage of people in a tropical country have this parasite in their systems is to examine their blood by night.

### Nocturnal Periodicity.

The most extraordinary feature about these embryos is that they appear in the peripheral blood only at night; they disappear entirely through the day, a phenomenon which is known by the name of filarial periodicity. Autopsies on people who have died by day have revealed the fact that the embryos are at this time packed in the lungs and large vessels of the thorax, but how they can keep their hold here against the blood stream until they choose again to pass out to the periphery is a point on which no explanation has yet been offered; all one can say is that it is a fact. On account of this it is at once evident that to demonstrate them one must take samples of night blood only, preferably between 9 p.m. and 6 a.m., and the technique employed for this is quite different from that of ordinary blood examination.

### Technique of Blood Examination.

As the filariæ are relatively very large (when, for example, they are compared with a malarial parasite), four or five large drops of blood are taken, placed on a slide and then spread out with a needle and allowed to dry in the air. After this the slide is placed in water to wash out the hæmoglobin of the red corpuscles, the filariæ and leucocytes remaining behind. On staining with fuchsin, methylene blue, or hæmotoxylin, and examining with a low power of the microscope the microfilariæ are easily detected. A big drop of blood may be examined fresh if the infection is a large one. This method is useful for studying movement and various points in the anatomy.

